# A cluster randomised trial of a telephone-based intervention for parents to increase fruit and vegetable consumption in their 3- to 5-year-old children: study protocol

**DOI:** 10.1186/1471-2458-10-216

**Published:** 2010-04-28

**Authors:** Rebecca J Wyse, Luke Wolfenden, Elizabeth Campbell, Leah Brennan, Karen J Campbell, Amanda Fletcher, Jenny Bowman, Todd R Heard, John Wiggers

**Affiliations:** 1 School of Medicine and Public Health, University of Newcastle, Newcastle, Australia; 2 Hunter New England Population Health, Newcastle, Australia; 3 School of Psychology and Psychiatry, Monash University, Melbourne, Australia; 4 Centre for Physical Activity & Nutrition Research, School of Exercise & Nutrition Sciences, Deakin University, Melbourne, Australia; 5 School of Psychology, University of Newcastle, Newcastle, Australia

## Abstract

**Background:**

Inadequate fruit and vegetable consumption in childhood increases the risk of developing chronic disease. Despite this, a substantial proportion of children in developed nations, including Australia, do not consume sufficient quantities of fruits and vegetables. Parents are influential in the development of dietary habits of young children but often lack the necessary knowledge and skills to promote healthy eating in their children. The aim of this study is to assess the efficacy of a telephone-based intervention for parents to increase the fruit and vegetable consumption of their 3- to 5-year-old children.

**Methods/Design:**

The study, conducted in the Hunter region of New South Wales, Australia, employs a cluster randomised controlled trial design. Two hundred parents from 15 randomly selected preschools will be randomised to receive the intervention, which consists of print resources and four weekly 30-minute telephone support calls delivered by trained telephone interviewers. The calls will assist parents to increase the availability and accessibility of fruit and vegetables in the home, create supportive family eating routines and role-model fruit and vegetable consumption. A further two hundred parents will be randomly allocated to the control group and will receive printed nutrition information only. The primary outcome of the trial will be the change in the child's consumption of fruit and vegetables as measured by the fruit and vegetable subscale of the Children's Dietary Questionnaire. Pre-intervention and post-intervention parent surveys will be administered over the telephone. Baseline surveys will occur one to two weeks prior to intervention delivery, with follow-up data collection calls occurring two, six, 12 and 18 months following baseline data collection.

**Discussion:**

If effective, this telephone-based intervention may represent a promising public health strategy to increase fruit and vegetable consumption in childhood and reduce the risk of subsequent chronic disease.

**Trial registration:**

Australian Clinical Trials Registry ACTRN12609000820202

## Background

Inadequate fruit and vegetable consumption contributes to a variety of chronic diseases and is estimated to be responsible for 2.6 million deaths per year worldwide [1]. A substantial proportion of adults [2,3] and children [4] from developed countries, including Australia [5,6], consume insufficient quantities of fruit and vegetables. The 2002 World Health Report estimated that 4% of the disease burden in developed countries was attributable to low fruit and vegetable intake [7]. Increasing consumption in early childhood may be an effective strategy to reduce the risk of subsequent chronic disease associated with insufficient fruit and vegetable consumption, as dietary patterns in childhood appear to track into adulthood [8].

Parents are likely to be influential in the development of children's eating behaviours [9]. Parental practices associated with increased child consumption of fruit and vegetables include increasing the availability and accessibility of fruit and vegetables within the home [10], role-modelling fruit and vegetable consumption [11] and establishing family eating routines supportive of fruit and vegetable consumption, such as eating meals as a family [12] not in view of a television [13]. Despite such influence, a lack of knowledge and skills can prevent parents from utilising these opportunities to promote healthy eating habits in their children [14].

Assisting parents to create supportive home environments can be an effective strategy to increase the fruit and vegetable consumption of their children [15]. However, studies involving traditional means of delivering interventions to parents, such as education sessions, often report high drop-out rates [16] and low attendance due to barriers associated with transport, work schedules and lack of interest [17]. Parent participation in healthy eating interventions is also reportedly constrained by specific barriers associated with preschool-aged children, including unpredictable sleep times and frequent sickness [18]. Telephone-based interventions may overcome many of these barriers and provide a convenient and effective means for parents to receive healthy eating support for their children. For example, previous research with adults has found that telephone support is an acceptable method of delivering health information [19] and is an effective strategy in modifying a range of health behaviours, including smoking [20], physical activity [21] and diet [22-24]. Furthermore, almost all Australian households have telephones [25]; thus, telephone-delivered interventions have the capacity for broad reach, and may hold promise in specifically targeting disadvantaged communities [26].

Despite the potential of telephone-based interventions to provide effective and acceptable support to parents, the authors are not aware of any randomised controlled trials of such interventions specifically targeting healthy eating behaviours in preschool children. The study attempts to address this gap in evidence through the conduct of a cluster randomised controlled trial of a telephone-based parent-focused intervention to increase the fruit and vegetable consumption of children aged 3 to 5 years. This paper describes the methodology to be employed in the conduct of this trial.

## Methods/Design

### Study Aim

The aim of this study is to examine the efficacy of a four-week telephone-based parent intervention in increasing fruit and vegetable consumption of 3- to 5-year-old children, as assessed by parental report.

### Study Design

#### Overview of study design

**Figure 1:**
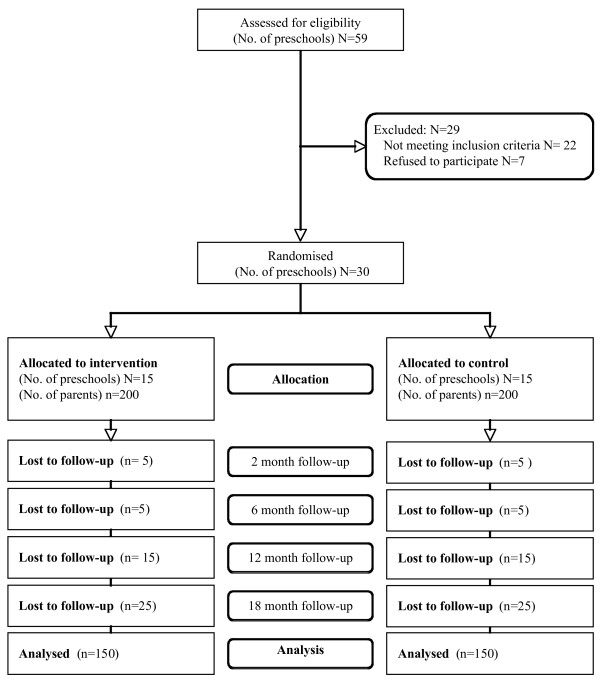
**CONSORT flow diagram estimating the progress of preschools and parents through the trial**.

The study employs a cluster randomised design, as outlined in Figure 1. The research will be reported in accordance with the requirements of the CONSORT statement [27]. Parents of 3- to 5-year-old children attending randomly selected preschools in the Hunter region of New South Wales, Australia, are being approached to participate. Preschools will be randomised to either control or intervention groups using a random number function in Microsoft Excel. Parents of children attending preschools allocated to the intervention group will receive a series of instructional resources and four 30-minute telephone calls delivered weekly by trained telephone interviewers. Parents of children attending preschools allocated to the control group will receive a readily available nutrition resource published by the Australian Government [28]. To assess the efficacy of the intervention, surveys will be conducted with parents *via *Computer Assisted Telephone Interview (CATI) at baseline (occurring one to two weeks prior to commencement of intervention delivery) and two, six, 12 and 18 months following baseline data collection.

The trial is funded by the Cancer Institute New South Wales (Ref no. 08/ECF/1-18). In-kind support for the trial is also provided by the Hunter New England Population Health Service. The trial has been approved by the Human Research Ethics Committees of the University of Newcastle (Ref No. H-2008-0410) and the Hunter New England Area Health Service (Ref No. 08/10/15/5.09).

### Research Setting

The study region encompasses non-metropolitan 'major cities' and 'inner regional' areas as described by the Australian Standard Geographic Classification system [29]. The region has lower indices of socio-economic status than the national average and has 485,700 residents, with 18,200 children aged 3 to 5 years [29]. Nine percent of Hunter residents speak languages other than English [30].

### Participants and Research Eligibility

#### Preschools

Thirty preschools will be recruited into the trial. Preschools in Australia provide educational and developmental programs for children (3 to 5 years) for up to two years prior to the commencement of full-time primary school education [31]. Preschool services are usually provided by qualified teachers for approximately six hours per weekday [32]. Sixty-four percent of all 4-year-old children in New South Wales attend preschool, with an average attendance of 17 hours per week [33]. Each preschool in the study area provides, on average, care for 27 children per day [34].

A current list of all preschools in the region that are licensed to provide care for 3- to 5-year-old children will be obtained from the New South Wales Department of Community Services (the licensing agency). Preschools will be excluded from the trial if they provide meals to children in their care (as this limits parents' capacity to influence the foods their children consume), cater exclusively for children with special needs (given the specialist care required for such children), are Government preschools (as conduct of the research has not been approved by the New South Wales Government Department of Education and Training) or have participated in child healthy eating research projects within six months of the commencement of recruitment. Information regarding eligibility of preschool services will be confirmed by preschool supervisors during phone contact as part of the recruitment process.

#### Parents

Four hundred parents will be recruited to the study. To be eligible, each participant must be a parent of a child aged 3 to 5 years attending a participating preschool, must reside with that child for at least four days a week (in order for the child to be sufficiently exposed to the intervention strategies that the parent may implement), must have some responsibility for providing meals and snacks to that child, and must be able to understand spoken and written English. Information regarding parent eligibility will be ascertained from completed study consent forms and verified during phone contact with parents immediately prior to baseline data collection. Parents will be excluded from the trial if their children have special dietary requirements or allergies that would necessitate specialised tailoring of the intervention or that may be adversely affected by the intervention. Such exclusions will be determined by an Accredited Practising Dietitian who is independent of the research team.

### Recruitment and Allocation

#### Preschools

Prior to formal requests to participate, the research trial will be promoted to preschools within the region through existing networks established by the *Good for Kids. Good for Life *program, a high-profile childhood obesity prevention program in the region [35]. Agreement has been reached with the *Good for Kids *program for this research project to utilise the *Good for Kids *brand and the program's communication channels with preschools. Specifically, newsletters and program emails will be used to make preschool supervisors aware of the trial and of what will be required of them if they consent to participate.

Participating preschools and the order in which they are to be approached to participate will be randomly selected from the New South Wales Department of Community Services database by an independent statistician using a random number function in Microsoft Excel. Recruitment will be staggered over a four- to five-month period due to intervention delivery capacity constraints. Preschools will therefore be approached in batches, until the desired sample of parents is achieved. The supervisors of the selected preschools will be sent letters and consent forms informing them of the study and requesting permission to recruit parents through their services. Consent will be obtained when the supervisor faxes or posts the consent form back to the research team. Two weeks after the initial information letters are sent to supervisors, a study research assistant will telephone supervisors who have not yet returned their consent forms to answer any questions they may have and to remind them to return their forms, confirming their consent or otherwise. Similar recruitment methods employed by the researchers as part of an Australian healthy eating and physical activity study were successful in achieving a childcare service participation rate of 84% [34].

#### Parents

In order to maximise parent participation in the study, a recruitment strategy based on a review of successful recruitment practices within the school setting [36] has been devised. Recruitment will incorporate the following four strategies recommended to maximise research participation.

##### 1. Recruitment oversight

One member of the research team will act as a dedicated recruitment coordinator. All preschool supervisors and parents will be provided with the direct phone number of the coordinator for all enquiries regarding research participation. The coordinator will also manage the rate at which preschools are recruited and monitor preschool and parent consent form return rates. The recruitment coordinator will not be involved in the delivery of the telephone support or the collection of data.

##### 2. Promotion of the research prior to requests for participation

A promotional flyer explaining the study will be sent to supervisors to disseminate to all parents at consenting preschools. The flyer will inform parents of the trial and the opportunity to participate, and will include endorsement of the research by a clinical psychologist and parenting expert. Such contact prior to a formal request to participate has been shown to increase response rates to postal questionnaires [37] and will be important in engaging parents where face-to-face contact is not possible. The project name, flyer and recruitment documentation will include the *Good for Kid*s logo and brand name [35]. Following a recent media campaign, unpublished data indicated that 59% of parents within the area reported that they were aware of the *Good for Kids *program.

##### 3. Dissemination of recruitment materials via methods to maximise parent engagement

The recruitment coordinator will arrange for recruitment packs to be delivered to each participating preschool, enough for one per family of each enrolled child aged 3 to 5 years. Distribution of these packs to parents will occur *via *methods considered by the preschool supervisor to be most effective and appropriate in engaging parents. Where possible, research staff will attend the preschool, hand out recruitment packs to parents and be available to answer parent questions. The recruitment pack consists of an information sheet, a consent form and a return envelope. The pack is brightly coloured and specifies that the study is being conducted in conjunction with a university; these strategies are suggested to increase response rates among those parents who have only received written communication during recruitment [37].

##### 4. Parent reminders

One to two weeks after delivery of the recruitment packs, reminder letters will be disseminated to parents, reminding them of the study and the opportunity to participate.

Parents will be asked to return the consent forms in the envelopes provided and place them in drop-boxes at their children's preschools within three weeks. The consent form includes a brief set of questions to establish the child's usual fruit and vegetable consumption. In order to identify any bias due to selective non-participation, all parents of 3- to 5-year-old children will be encouraged to complete the items on the consent forms and return them, regardless of whether they choose to participate.

#### Random allocation of preschools

Following the recruitment of parents within a preschool, an independent statistician will randomly allocate the preschool to an intervention or a control group using a randomisation function in Microsoft Excel. Randomisation at the unit of the preschool, rather than the individual parent, will reduce the potential for intervention contamination between parents whose children attend the same preschool [38]. Based on evidence suggesting that children's eating environments differ by socio-economic status [39], the randomised allocation will be stratified by the socio-economic status of the area in which the preschool is located [40]. Preschools with a postcode in the top 50% of the state, based on Socio-Economic Indexes for Areas (SEIFA) [41] will be defined as 'high socio-economic area preschools' and those within the lower 50% will be defined as 'low socio-economic area preschools'. Preschools will be randomised in a 1:1 (intervention:control) ratio in randomly sequenced blocks of between two and six preschools. Block randomisation will maximise the likelihood that the number of participants allocated to each group remains approximately equal [42]. Due to the difficulty in concealing group allocation from participants, parents will not be blinded, and following baseline data collection they will receive letters informing them that they will receive either print materials or telephone support.

### Intervention Group

The 200 parents randomised to the intervention group will receive a workbook and other resources and weekly scripted telephone contacts of approximately 30 minutes' duration delivered over four weeks. Telephone-based interventions of a similar intensity have previously been found to be effective in adults [43,44]. Each telephone contact aims to provide parents with appropriate knowledge and skills to modify three key domains within the home food environment: availability and accessibility of fruit and vegetables; supportive family eating routines, and parental role-modelling (See Table 1).

**Table 1:** Overview of intervention call content: behaviour change techniques and their application

Key Theme	Content	Behaviour Change Technique	Application of Behaviour Change Technique
**WEEK 1** Availability and Accessibility	Dietary recommendations and serving sizes		
	Children's food diary	Prompt self-monitoring of behaviour	Parents are asked to monitor their children's intake of fruit, and vegetables over 3 days.
	Ways to provide fruit and vegetables throughout the day		
	Setting goals	Prompt specific goal-setting	Parents are encouraged to set a program goal.

**WEEK 2** Availability and Accessibility, Supportive Family Eating Routines	Changing the family routine	Prompt intention formation	Parents decide which activities they will attempt in the coming week.
	Availability and accessibility of foods in the home	Provide general encouragement	Interviewers provide positive feedback about any helpful practices occurring in the home.
	Mealtime practices	Teach to use prompts or cues	Parents learn the HELPS acronym, i.e. try to eat when Hungry, not attempting anything else at the same time (focus on Eating), at an appropriate Location to eat, from a Plate, and while Sitting.
	Meal planning		
	Review of goals	Prompt review of behavioural goals	Parents review the goals they set during the previous calls and evaluate their progress.

**WEEK 3** Parental role-modelling, Supportive Family Eating Routines	The Ps and Cs division of feeding responsibility	Teach to use prompts or cues	Parents learn the Ps and Cs: Parents are encouraged to Plan, Prepare and Provide. Children are encouraged to Choose (whether, what and how much to eat) [49].
	Mealtime strategies to encourage vegetable consumption	Prompt intention formation	Parents decide which activities they will attempt in the coming week.
		Provide general encouragement	Interviewers provide positive feedback about any helpful practices occurring in the home.
	Role-modelling of fruit and vegetable consumption	Prompt identification as a role model	Parents are provided information about their importance in role-modelling fruit and vegetable consumption. Their consumption is compared with national nutrition recommendations. Tailored feedback is provided.

**WEEK 4** Availability and Accessibility Parental role-modelling, Supportive Family Eating Routines	Review of weeks 1-3	Provide general encouragement	Interviewers provide positive feedback about any helpful practices occurring in the home
	Planning for the future and dealing with difficult situations	Prompt barrier identification	Parents are encouraged to identify barriers that will prevent them implementing what they have learnt and to generate solutions.
	Review of goals	Prompt review of behavioural goals	Parents review their program goal, evaluate their progress and identify how they can maintain the change

#### Development and pre-testing of the intervention

The script has been developed by an expert advisory group of clinical and health psychologists, dietitians and health promotion practitioners. The script utilises CATI software [45] to tailor support based on parental report of the home food environment. Intervention development was guided by an existing framework for behavioural therapy development in clinical settings [46]. The pre-testing process involved three phases where the research team piloted preliminary versions of the telephone script and workbook, and refined the intervention based on the feedback received. Each phase of pre-testing was conducted with eight to 12 volunteer health promotion practitioners, parenting experts and parents of young children. Volunteers were asked to comment on content, structure, presentation and length of the intervention, and were encouraged to suggest how the telephone script or workbook could be improved. Feedback from the members of the research team who administered the pre-test telephone calls to volunteers was also sought regarding the ease of administration of the script and the level of volunteer engagement in the intervention.

Following each pre-testing phase, feedback was collated and proposed intervention amendments were discussed by the research team and adopted where feasible. The primary amendments to the intervention telephone script resulting from pre-testing included reducing the length of the calls; changing the order of presentation of intervention content; reducing repetition; providing more examples to clarify key issues; simplifying language; removing jargon; making the script more conversational; and including more opportunities for interaction between parents and interviewers. The primary amendments to the workbook included the addition of more practical information and tools for parents, improving readability through simplifying language, using subheadings and reducing the volume of text, and improvements to the presentation of the workbook to make it more appealing, such as use of bright colours, illustrations and photographs.

#### Intervention content

The telephone intervention script is designed to help parents modify their home food environments through addressing three key domains listed in Table 1. The first column of the table lists each domain at the point at which it appears in the schedule of support calls, while the second column lists the specific topics that are used to explore each of the given domains. Each domain has been associated with increased fruit and vegetable consumption in children as described below.

##### a) Availability and accessibility of fruit and vegetables [10,47]

The telephone intervention encourages parents to ensure that fruit and vegetables are available and accessible in the home and that they are prepared, presented or maintained in a ready-to-eat form that encourages their consumption [48]. This could include offering cut-up pieces of fruit or vegetable at snack times, and ensuring fruit is visible by storing it in fruit bowls.

##### b) Supportive family eating routines

The intervention will seek to improve parent knowledge and facilitate the acquisition of skills to support parents to eat meals as a family [12] without the television on [13], establish and enforce family rules about eating [11] and develop boundaries regarding when and how food is offered to their children [49].

##### c) Parental role-modelling of fruit and vegetable consumption [11]

Parents will be encouraged to increase the number of serves of fruit and vegetables that they consume in front of their children and to express supportive attitudes toward the consumption of fruit and vegetables to their children, for example, by making positive and encouraging comments.

Participants will also be asked to undertake homework activities to encourage them to apply, directly into their home environment, the strategies and information covered in the telephone calls. Incorporating homework assignments into health behaviour interventions has been found to increase the size of the intervention effect [50]. Homework activities will be optional and tailored to the needs of the participant, based on recommended home food environment practices not currently undertaken by the participant.

#### Intervention resources

Based on evidence indicating telephone-based dietary interventions are more effective when used in conjunction with print and other resources [21], all intervention participants will be mailed resource kits following completion of the baseline survey. The kit comprises a participant workbook containing information and activities, a pad of meal planners, and a cookbook including recipes high in fruit and vegetables. The resources will be used to facilitate participant engagement in the telephone support calls and assist participants to complete intervention activities between telephone contacts.

#### Conceptual model

The telephone-based intervention accords with the model of family-based intervention proposed by Golan and colleagues [51] in the treatment and prevention of childhood obesity. Their model, which draws upon socio-ecological theory, focuses on introducing new familial norms associated with healthy eating. This is achieved through making changes within the home food environment, providing positive parental role-modelling and increasing parenting- and nutrition-related knowledge and skills. Interventions based on such a model have been shown to be effective in bringing about environmental changes in participants' homes to support healthy eating [52] and in reducing poor eating habits of overweight and obese children of participants [53].

The intervention utilises a number of specific behaviour change techniques to initiate the change process as describes in Table 1. The third column lists the behaviour change techniques used and the fourth column links each technique to its application in the context of the topic listed in column 2. These behaviour change techniques include prompting intention formation, barrier identification, specific goal-setting and the reviewing of such goals, self-monitoring of behaviour and identification as a role-model, teaching to use prompts or cues, and providing general encouragement, as described in the taxonomy proposed by Abraham and Michie [54].

#### Intervention personnel, recruitment and training

Consistent with other telephone-based health behavioural interventions [20,21], intervention support will be delivered by trained telephone interviewers. Interviewers delivering the intervention will have experience in conducting health-related telephone surveys, but have no formal qualifications in psychology, dietetics, parenting, health promotion or other health professions. The use of telephone interviewers without specialist skills may mean that adoption of this intervention by government agencies is more feasible. Interviewers without specialist skills have previously been found to be effective in improving other health behaviours [20]. If effective in this context, their use may facilitate the adoption of this type of intervention where use of specialist staff may not be feasible due to cost and the shortage of staff with such skills.

To recruit suitable staff and to equip them with the necessary knowledge and skills to deliver the intervention, a pool of potential telephone interviewers was invited to attend a two-day training workshop. The training was developed and delivered by a registered dietitian, a clinical psychologist specialising in parenting, and health promotion practitioners (with post-graduate qualifications and experience in public health). The research team and clinical psychologist judged interviewer competency, based on the completion of role-plays [55] and small group exercises during training, and those considered sufficiently competent were selected to deliver the intervention. The selected interviewers were then required to complete a further minimum 10 hours of self-paced practice, including script and workbook familiarisation. They were also required to practise each script with a member of the research team to ensure that required levels of competency and adherence had been met [55] and that they were able to deliver the script in a confident, conversational style and respond appropriately to participant queries.

During the first two months of intervention delivery, all interviewers will participate in fortnightly group supervision, facilitated by a psychologist. A self-regulatory model of peer supervision [56] will be utilised to facilitate learning, improve interviewer performance and help standardise intervention delivery. Members of the research team will monitor the supervision sessions and provide feedback as required.

#### Intervention monitoring

To ensure integrity of intervention delivery during the trial, members of the research team will have weekly contact with interviewers to keep abreast of common issues and concerns so that they may be addressed in a consistent manner. During each four-week batch of telephone calls, members of the research team will monitor at least two completed calls made by each interviewer to assess adherence with the intervention protocol. Specifically, the research team member will record whether the interviewer covers the key themes and information for each call, the extent to which the interviewer deviates from the script, the length of the call and whether the interviewer adequately answers any questions asked.

The records of the recruitment coordinator will be audited following the recruitment of each batch of participants. A separate member of the research team will review the dates on which allocation letters are mailed. They will also review the attempt dates, receipt dates and completion dates of intervention and data collection telephone calls for each trial participant. This periodic review of documentation will assess whether the intervention is progressing in a timely manner and in accordance with the study protocol [57].

### Control Group

Participants allocated to the control group will receive a 22-page booklet, 'The Australian Guide to Healthy Eating: Background information for consumers' [28]. This is a national food guide published by the Australian Government Department of Health and Ageing. This publication will be posted to parents' residential addresses following completion of the baseline survey.

### Data Collection and Measures

Baseline and follow-up data will be collected through a CATI survey administered to all participants. The survey will take approximately 30 minutes to complete. Data collection interviewers will be provided with training to ensure that they understand and adhere to data collection protocols, and to practise the survey script.

Baseline data will be collected one to two weeks prior to intervention delivery. Calls will be monitored for adherence to the training protocol. Members of the research team will monitor approximately ten percent of the first batch of baseline calls and compare the delivery of the survey to the script as written. Any deviations from the protocol will be addressed with the interviewer immediately following the completion of the call. Each interviewer will then be monitored at least once in each subsequent batch of surveys to ensure consistency over time. The survey administered at baseline will be repeated at four time points: two, six, 12 and 18 months following baseline data collection. To minimise attrition, prior to follow-up data collection calls at six, 12 and 18 months, participants will receive letters thanking them for their participation to date and reminding them that they will shortly be telephoned to participate in follow-up phone calls [58].

Data collection interviewers will not participate in trial recruitment or intervention delivery and will be blind to participant group allocation. Furthermore, during each follow-up data collection interview, participants will be asked not to disclose their group allocation to the interviewers at the start of the telephone survey. To assess the effectiveness of the blinding, following the collection of trial outcome data, interviewers will be asked to nominate the groups to which they believe the participants were allocated [59].

### Measures

#### Demographics

Demographic items regarding parents' gender, age, Aboriginal and/or Torres Strait Islander status, education, income, postcode and household composition (e.g. the number of children in the household), as well as questions regarding the child's gender and age, will be assessed at baseline. Items used to assess demographics will be sourced from the NSW Health Survey Program, a regular government behavioural risk factor surveillance survey [60].

#### Process measures

The CATI system will record information regarding the outcome of each attempted call (e.g. engaged, answering machine, call-back arranged, call partially complete, call complete or refusal), the interviewer who attempted the call, the date and time of the attempt, the call duration and the responses provided by the participant throughout the call. During intervention delivery calls, participants will be asked whether they received the intervention resources and what homework activities they attempted. This will allow for an assessment of the extent to which the intervention was delivered and received as planned.

#### Primary outcome measure: fruit and vegetable consumption

The primary outcome is the change in the fruit and vegetable intake of the preschool children. Fruit and vegetable intake will be assessed using the fruit and vegetable subscale of the Children's Dietary Questionnaire. This questionnaire was developed to assess Australian children's dietary patterns in relation to current national guidelines and has been recommended for use in assessing the efficacy of interventions to improve children's eating habits [61].

This semi-quantitative food frequency questionnaire asks parents to report the frequency and variety of foods consumed by their children over the previous seven days and the previous 24 hours. Scores on the fruit and vegetable subscale range from 0 to 28, with a score of 14 recommended based on current national dietary guidelines [61]. A one-point increase on this subscale could equate to, for example, a child consuming on average an additional type of fruit or vegetable each day (variety), or consuming fruit or vegetables at an additional eating occasion each day (frequency). An increase of this magnitude of fruit and vegetable variety or frequency of consumption is consistent with effect sizes of fruit and vegetable consumption reported in previous child fruit and vegetable interventions, and has the potential to have significant public health impact [62]. Reliability and validity of this tool has been established using multiple samples of Australian children, including preschoolers [61]. The fruit and vegetable subscale was found to be internally consistent (∝ = 0.76), reliable (intra-class correlation coefficient = 0.75) and valid as assessed against a seven-day food checklist (Spearman's correlation coefficient = 0.58) [61].

#### Sample size

A sample size of approximately 300 participants (150 per group) at the 18-month follow-up will allow a detectable difference between intervention and control groups of 1.27 on the fruit and vegetable subscale of the Children's Dietary Questionnaire, with 80% power at the 0.05 significance level. This sample size accounts for the effect of clustering by assuming an interclass correlation coefficient of 0.03 (unpublished data from the *Good for Kids *program) and assumes 10 participants per preschool remain at the 18-month follow-up (as explained below).

Four hundred participants will be required to be recruited at baseline to achieve the desired sample of 300 at the 18 month follow-up. Based on preschools caring for an average of 27 children each day [34], and assuming children attend preschool for an average of 2.8 days per week (i.e. 17 hours over 6-hour long days), it is expected that up to 48 parents of children, on average, will be eligible to participate in the trial from each consenting preschool. A parent participation rate of 30% [19] will yield approximately 14 parents per preschool at baseline, of whom 10 will remain at 18 months, assuming a 25% attrition rate [63]. It is thus estimated that 30 preschools will be required to generate a sample of 300 parents at the conclusion of the trial.

#### Statistical analysis: primary outcome

All statistical analyses will be performed with SAS (version 9.2 or later) statistical software.

To assess the initial impact of the intervention and the extent to which any intervention effect is maintained in the longer term, the primary outcome analyses for the trial will be conducted on participant scores on the fruit and vegetable subscale of the Children's Dietary Questionnaire collected at the two-month and 18-month follow-up time periods. For the primary outcome analyses, an alpha value of 0.05 will be utilised to determine statistical significance.

Outcome data will be analysed using general estimating equations based on the intention-to-treat principle, where participants are analysed based on the groups to which they were allocated, regardless of the treatment type or exposure that they actually received [64]. General estimating equation models will account for any clustering effect of preschools. To ensure the results of the primary analysis are robust against the missing data assumption of the general estimating equation, a sensitivity analysis will be performed whereby participants' observations at baseline will be used as a substitute for subsequent missing data. A per-protocol analysis will also be conducted whereby outcome data will only be included in analyses if participants received and completed all four telephone support calls. Conducting both intention-to-treat and per-protocol analyses is recommended when assessing trial outcomes [64].

## Discussion

To the authors' knowledge, this is the first randomised controlled trial to evaluate a telephone-based parent intervention to increase the fruit and vegetable intake of preschool-aged children. The intervention has been developed to maximise the likelihood of having a positive effect on fruit and vegetable consumption through the use of a relevant conceptual model during intervention development, and employing specific behaviour change strategies to target characteristics of the home food environment known to be associated with increased fruit and vegetable intake.

The study demonstrates many strengths: the experimental randomised design; the implementation of procedures to reduce potential threats to internal validity, such as the blinding of data collection interviewers and computer-based randomisation of groups undertaken by an independent statistician; the use of an outcome measure with established validity and reliability; and the recruitment of study participants from a setting which most 4-year-old children attend on multiple days of the week. If found to be effective, an intervention of this intensity, utilising trained staff rather than experienced health professionals, is considered to have the potential to be implemented on a community-wide basis, as currently exists for adult risk behaviours [20].

## Conclusion

This manuscript provides a comprehensive description of the study methods to be employed as part of a randomised controlled trial of a telephone-based parent intervention to increase the fruit and vegetable intake of children aged 3 to 5 years. The successful implementation of this trial will provide strong evidence on which to base judgements regarding the efficacy of this intervention approach.

## Competing interests

The authors declare that they have no competing interests.

## Authors' contributions

Author RW led the development of this manuscript. Author LW conceived the intervention concept and secured the funding source. Authors RW, AF, LW and LB contributed to the development of the intervention scripts and printed material. Authors LW, KC, RW and JW determined the measures to be used and the analyses to be conducted. All authors contributed to the research design and trial methodology and contributed to, read and approved the final version of this manuscript.

## Pre-publication history

The pre-publication history for this paper can be accessed here:

http://www.biomedcentral.com/1471-2458/10/216/prepub
